# Effects of Body Mass Index and Body Weight on Plasma Concentration of Ticagrelor and Platelet Aggregation Rate in Patients with Unstable Angina in a Chinese Han Population

**DOI:** 10.31083/j.rcm2503083

**Published:** 2024-03-04

**Authors:** Houling Guo, Qingqi Li, Fei He, Cheng Cheng, Min Wang, Banglong Xu, Xiaochen Wang, Jianlong Sheng

**Affiliations:** ^1^Department of Cardiology, The Second Affiliated Hospital of Anhui Medical University, 230601 Hefei, Anhui, China

**Keywords:** platelet aggregation rate, unstable angina, body mass index, ticagrelor, dual antiplatelet therapy

## Abstract

**Background:**

The aim of this study was to investigate the impact of body mass index (BMI) and body weight on the concentrations of ticagrelor and the ticagrelor metabolite, AR-C124910XX, as well as the platelet aggregation rate (PAR) in a Chinese Han population with unstable angina (UA). Specifically, it focused on these parameters following the administration of dual antiplatelet therapy (DAPT) comprising aspirin and ticagrelor.

**Methods:**

A total of 105 patients with UA were included in the study. Measurement of the platelet aggregation rate induced by adenosine diphosphate (PAR-ADP) was performed before, as well as 3 and 30 days after DAPT treatment. The plasma concentrations of ticagrelor and AR-C124910XX were detected at 3 and 30 days after DAPT treatment. We conducted correlation analyses to assess the effects of BMI and body weight on the concentrations of ticagrelor and AR-C124910XX, on PAR-ADP, and on the inhibition of platelet aggregation induced by adenosine diphosphate (IPA-ADP) at both 3 and 30 days after DAPT treatment.

**Results:**

The BMI and body weight were positively correlated with baseline PAR-ADP (*r* = 0.205, *p* = 0.007; *r* = 0.122, *p* = 0.022). The PAR-ADP at 3 and 30 days after DAPT treatment were significantly lower than at baseline (61.56% ± 10.62%, 8.02% ± 7.52%, 12.90% ± 7.42%, *p* < 0.001). There was a negative correlation between body weight and the concentrations of ticagrelor and AR-C124910XX at 3 days following DAPT treatment (*r* = –0.276, *p* < 0.001; *r* = –0.337, *p* < 0.001). Additionally, BMI showed a similar negative correlation with the concentrations of ticagrelor and AR-C124910XX (*r* = –0.173, *p* = 0.009; *r* = –0.207, *p* = 0.002). At 30 days after treatment, both body weight and BMI were negatively correlated with ticagrelor (*r* = –0.256, *p* < 0.001; *r* = –0.162, *p* = 0.015) and its metabolite (*r* = –0.352, *p* < 0.001; *r* = –0.202, *p* = 0.002). Body weight was positively correlated with PAR-ADP (*r* = 0.171, *p* = 0.010) and negatively correlated with IPA-ADP (*r* = –0.163, *p* = 0.015) at 30 days after treatment. Similarly, BMI was positively correlated with PAR-ADP (*r* = 0.217, *p* = 0.001) and negatively correlated with IPA-ADP (*r* = –0.211, *p* = 0.001) at the same time point.

**Conclusions:**

BMI and body weight are key factors influencing the pharmacokinetics and pharmacodynamics of ticagrelor in Chinese Han patients with UA following DAPT treatment that includes ticagrelor. Both BMI and body weight were positively correlated with PAR-ADP at baseline and 30 days after DAPT treatment.

**Clinical Trial Registration:**

ChiCTR2100044938, https://www.chictr.org.cn/.

## 1. Introduction

Dual antiplatelet therapy (DAPT), combining aspirin with a P2Y12 receptor inhibitor (or adenosine diphosphate [ADP] receptor blocker), is essential for treating acute coronary syndrome (ACS) patients after percutaneous coronary intervention (PCI) [1]. Research, notably the PLATO study [1], demonstrates that aspirin and ticagrelor—a potent P2Y12 receptor inhibitor—enhance ACS prognosis more effectively than aspirin and clopidogrel. While ticagrelor exerts a stronger inhibitory effect on platelet aggregation than clopidogrel, which can further reduce coronary ischemic events, the treatment is associated with an increased risk of bleeding [2, 3]. The patient response to DAPT treatment may vary, with a high responders facing greater bleeding risk, and low responders higher ischemia risk [4]. Optimizing ticagrelor’s use, either by mitigating bleeding risk factors or tailoring doses to individual responses, could reduce bleeding without increasing cardiovascular ischemic events.

Body mass index (BMI) and body weight significantly influence drug metabolism [5]. Previous studies have suggested a link between higher BMI and reduced efficacy of P2Y12 receptor inhibitors following clopidogrel treatment [6]. With ticagrelor’s increased use as a P2Y12 receptor inhibitor, its pharmacokinetics and pharmacodynamics in relation to BMI has undergone further investigation. Studies have reported a positive correlation between BMI and platelet reactivity [7]. Pharmacokinetic studies found that, compared with Caucasians, Chinese patients exhibit higher peak blood concentrations of ticagrelor and its metabolites after administration [8]. This finding is intriguing given the generally lower BMI of the Chinese population compared to Caucasians [9]. It raises questions about how BMI and body weight might affect serum concentrations of ticagrelor and its metabolites, and subsequently, its antiplatelet effects in Chinese patients with unstable angina (UA). Therefore, this study was undertaken to address these issues.

## 2. Methods

### 2.1 Study Subjects and Specimen Collection

#### 2.1.1 Study Subjects

This study recruited patients diagnosed with UA in the Department of Cardiology of the Second Affiliated Hospital of Anhui Medical University from September 2021 to June 2022. The UA diagnostic criteria were in accordance with published guidelines [10]. The exclusion criteria were as follows: (1) patients with severe infection, malignant tumors, rheumatic connective tissue disease, and hemoglobin <90 g/L; (2) patients receiving glucocorticoid therapy; (3) patients who received clopidogrel or ticagrelor within 1 week before this admission, and patients who discontinued ticagrelor within 3 days after receiving ticagrelor because of adverse drug reactions; (4) patients who received strong inhibitors (e.g., clarithromycin, ketoconazole, itraconazole) or inducers (e.g., tegretol, rifampicin, phenobarbital) of cytochrome P450 (CYP) 3A4 during the study. The study protocol was approved by the Ethics Committee of the investigators’ institution (YX2021-008). All patients or their family members provided signed written consent to participate in the study. This study investigated the impact of BMI and body weight on the pharmacokinetics and pharmacodynamics of ticagrelor by correlation analysis in patients diagnosed with UA. The sample size (α = 0.05, β = 0.2, *r* = 0.2–0.8) was estimated using MedSCI Sample Size Tools (MSST.v5.9.5, MedSci Corp., Songjiang, Shanghai, China) software with at least 8 cases in this study. To improve the power of statistical testing, we planned to enroll not less than 100 subjects. Overall, a total of 105 UA patients were included in this study.

#### 2.1.2 Collection of Clinical Data

Concomitant diseases were recorded, including hypertension, cerebrovascular diseases (including transient ischemic attack, ischemic stroke, and hemorrhagic stroke), diabetes, smoking history (average >10 cigarettes/day, lasting for more than 1 year), and alcohol history (average >100 g/day, lasting for more than 1 year). The patient’s height and weight at admission were measured, and BMI (kg/$m^{2}$) was calculated.

#### 2.1.3 Baseline Specimen Collection and Drug Administration

Upon admission, peripheral venous blood samples were collected after admission and before treatment with ticagrelor. These samples were used to measure the platelet aggregation rate induced by ADP (PAR-ADP). After specimen collection, all patients received aspirin enteric-coated tablets (100 mg/tablet, lot number: BJ 55920, Bayer S.p.A., Viale Certosa, Milano, Italy). A loading dose of 300 mg was administered unless the patient had been on long-term aspirin therapy, in which case the loading dose was omitted, and a daily oral dose of 100 mg was continued. Additionally, ticagrelor (90 mg/tablet, lot number: 2008112, AstraZeneca AB, Gärtunavägen, Södertälje, Sweden) was administered with a loading dose of 180 mg, followed by a maintenance dose of 90 mg twice daily.

#### 2.1.4 Clinical Management

Guideline-recommended drug therapy was initiated, tailored to each patient’s specific condition [10], and PCI was performed based on criteria in accordance with PCI guidelines [11]. Patients were treated with DAPT containing ticagrelor for at least 12 months according to UA guidelines, provided there were no contraindications or adverse reactions.

#### 2.1.5 Specimen Collection at 3 and 30 Days after DAPT Treatment

Following DAPT treatment, venous blood was collected at both 3 and 30 days later (with a time window allowance of ±3 days for the 30-day collection). These samples were drawn in the morning before administering ticagrelor or PAR-ADP tests. Following collection, the plasma was separated and refrigerated at –70 °C, and the concentrations of ticagrelor and AR-C124910XX were detected at selected times. To calculate the inhibition of platelet aggregation induced by adenosine diphosphate (IPA-ADP) after treatment, we divided the difference in PAR-ADP levels at 3 and 30 days after DAPT treatment by the baseline PAR-ADP.

#### 2.1.6 Follow-Up

The follow-up was completed through outpatient clinics, WeChat, Internet hospitals, or telephone follow-up. During the follow-up period, major adverse cardiac events (MACEs) such as new acute coronary ischemia events, unplanned PCI, death, ischemic stroke, and clinically significant bleeding events were recorded. Clinically significant bleeding events were evaluated according to the bleeding classification criteria uniformly defined by the Bleeding Academic Research Consortium (BARC) [12] and were defined as bleeding with BARC type 2-5. Patients were followed up for up to 12 months after DAPT treatment, after which they transitioned to routine clinical follow-up.

### 2.2 Test Methods for Research Indicators

#### 2.2.1 Detection of PAR

PAR was detected using an AggRAM platelet aggregation meter and supporting reagents (Helena Laboratories, Beaumont, TX77704, USA, NO:8JF52001), using photoelectric turbidimetry to detect PAR-ADP. To prepare samples, whole blood was treated with anticoagulant (0.11 mmol/L citrate), and centrifuged at room temperature to obtain platelet-rich plasma (PRP) and platelet-poor plasma (PPP). The platelet aggregation in PRP was induced by ADP at a concentration of 20 µmol/L. The calculated maximum platelet aggregation is the PAR-ADP.

#### 2.2.2 Detection of the Concentrations of Ticagrelor and AR-C124910XX

Peripheral venous blood samples, with a volume of 4 mL, were drawn with an anticoagulant tube containing heparin. All samples were immediately soaked in an ice bath, and centrifuged at 1500 *g* centrifugal force within 0.5 h and at 4 °C for 10 min. Following these steps, the plasma was separated and refrigerated at –70 °C for testing. The high-performance liquid chromatography–tandem mass spectrometry method was used to complete blood concentration levels.

### 2.3 Statistical Analysis

Statistical analysis was conducted using SPSS 19.0 (IBM Corp., Armonk, NY, USA). The results of measurement data were expressed using the mean ± standard deviation ($\bar{x}$ ± SD) or median and interquartile range. The Analysis of Variance was performed to compare measurement data that was normally distributed among groups. The data non-normally distributed or non-uniform variance among groups was performed by non-parametric testing. PAR-ADP before and after DAPT treatment at 3 and 30 days was analyzed by paired-samples *t* test. Kendall’s correlation analysis was performed to analyze the correlation between BMI or body weight, and PAR-ADP, IPA-ADP, ticagrelor concentration, and AR-C124910XX concentration. Cox multivariate regression was employed to analyze factors influencing MACEs and bleeding events post-DAPT, including sex, age, histories of hypertension, diabetes, smoking, cerebrovascular disease, PAR-ADP, IPA-ADP, and ticagrelor and AR-C124910XX concentrations. A *p*-value < 0.05 was considered statistically significant.

## 3. Results

### 3.1 Clinical Indicators

A total of 105 patients were included in the study. Patients had a mean age 61.46 ± 10.48 years, including 76 males (72.4%), 74 patients (70.5%) with hypertension, 31 patients (29.5%) with diabetes, 44 patients (41.9%) with a smoking history, and 7 patients (6.7%) with a history of PCI. The average low-density lipoprotein cholesterol was 2.70 ± 1.07 mmol/L. All enrolled patients underwent PCI.

Statins were administered to 99 patients (94.3%), while six patients (5.7%) with hepatic dysfunction or statin intolerance did not receive statins. Treatment with β-Blockers was used in 58 patients (55.2%). Because of the risk of Due to gastrointestinal bleeding risk in patients treated with DAPT, proton pump inhibitors were administered to 84 (80.0%) patients.

### 3.2 Results of Body Weight, BMI, PAR-ADP, IPA-ADP, and the Plasma Concentration of the Drug

#### 3.2.1 Basic Data 

At baseline and 30 days after DAPT treatment, the mean body weight was 68.11 ± 11.91 and 68.01 ± 11.86 kg, respectively; the mean BMI at baseline and 30 days after DAPT treatment was 25.10 ± 3.20 and 25.08 ± 3.22 kg/$m^{2}$, respectively. The mean plasma concentrations of ticagrelor at 3 and 30 days after treatment were 557.82 ± 298.90 and 504.48 ± 199.65 ng/mL, respectively (*p* = 0.007). The mean concentration of the metabolite AR-C124910XX at 3 and 30 days was 265.96 ± 185.93 and 243.08 ± 111.28 ng/mL, respectively (*p* = 0.031), suggesting that the plasma concentration at 3 days after DAPT was significantly higher than that at 30 days. The values of PAR-ADP at 3 and 30 days after DAPT treatment were significantly lower than at baseline (61.56% ± 10.62%, 8.02% ± 7.52%, 12.90% ± 7.42, *p* < 0.001) (Table 1). In contrast, PAR-ADP at 30 days after DAPT treatment slightly increased compared with that at 3 days and the baseline level (*p* < 0.001). The IPA-ADP at 3 and 30 days reflecting the platelet aggregation inhibition intensity after DAPT treatment was 88.06% ± 10.81% and 78.71% ± 12.47%, respectively (*p* < 0.001).

**Table 1.** **The values of body weight, BMI, concentrations of ticagrelor and AR-C124910XX, PAR-ADP, and IPA-ADP**.

Characteristics	Baseline	3 days	30 days	*p *value
Body weight (kg)	68.11 ± 11.91	/	68.01 ± 11.86	0.037
BMI (kg/$m^{2}$)	25.10 ± 3.20	/	25.08 ± 3.22	0.560
Ticagrelor (ng/mL)	/	557.82 ± 298.90	504.48 ± 199.65	0.007
AR-C124910XX (ng/mL)	/	265.96 ± 185.93	243.08 ± 111.28	0.031
PAR-ADP (%)	61.56 ± 10.62	8.02 ± 7.52	12.90 ± 7.42	<0.001
IPA-ADP (%)	/	88.06 ± 10.81	78.71 ± 12.47	<0.001

Abbreviations: BMI, body mass index; PAR-ADP, platelet aggregation rate induced by adenosine diphosphate; IPA-ADP, inhibition of platelet aggregation induced by adenosine diphosphate.

#### 3.2.2 Sex-Based Analysis of Body Weight, BMI, Ticagrelor and AR-C124910XX Concentrations, and PAR-ADP and IPA-ADP Levels

Female patients exhibited lower body weight, BMI and baseline PAR-ADP compared to male patients. Following ticagrelor treatment, females had higher blood concentrations of ticagrelor and its metabolite AR-C124910XX. At 30 days following treatment, female patients showed reduced PAR-ADP levels but increased IPA-ADP levels compared to males (Table 2).

**Table 2.** **The values of body weight, BMI, concentrations of ticagrelor and AR-C124910XX, PAR-ADP, and IPA-ADP analyzed by sex**.

Characteristics		Male (n = 76)	Female (n = 29)	*p* value
Body weight (kg)				
	Baseline	72.16 ± 9.76	57.50 ± 10.54	<0.001
	30 days	72.05 ± 9.73	57.43 ± 10.41	<0.001
BMI (kg/$m^{2}$)				
	Baseline	26.56 ± 2.75	23.82 ± 3.99	0.004
	30 days	25.59 ± 2.78	23.80 ± 3.88	0.005
Ticagrelor (ng/mL)				
	3 days	488.72 ± 200.31	738.90 ± 421.25	0.003
	30 days	476.58 ± 180.30	577.62 ± 230.50	0.005
AR-C124910XX (ng/mL)				
	3 days	212.13 ± 104.83	407.73 ± 265.50	<0.001
	30 days	215.09 ± 90.64	316.41 ± 127.64	<0.001
PAR-ADP (%)				
	Baseline	64.53 ± 10.39	60.15 ± 11.37	0.019
	3 days	7.22 ± 7.33	10.10 ± 7.73	0.17
	30 days	15.00 ± 7.13	11.25 ± 8.25	0.032
IPA-ADP (%)				
	3 days	89.24 ± 10.65	84.96 ± 10.77	0.133
	30 days	72.49 ± 12.15	80.58 ± 13.47	0.037

Abbreviations: BMI, body mass index; PAR-ADP, platelet aggregation rate induced by adenosine diphosphate; IPA-ADP, inhibition of platelet aggregation induced by adenosine diphosphate.

#### 3.2.3 The Change From Baseline to 30-days in PAR-ADP by BMI Category 

**Fig. 1.**
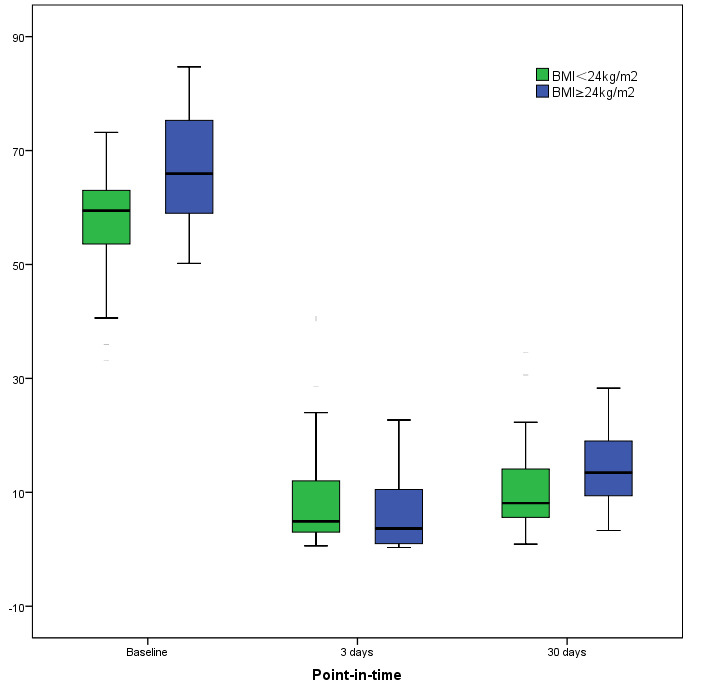
**The change from baseline to 30-days in PAR-ADP by BMI category**. BMI, body mass index.

Patients with a BMI ≥24 kg/$m^{2}$ are defined as overweight, so we divided the BMI category into a high BMI group (BMI ≥24 kg/$m^{2}$) and a low BMI group (BMI <24 kg/$m^{2}$). Patients in the high BMI group had a higher PAR-ADP at baseline and 30 days after treatment with ticagrelor (Table 3, Fig. 1).

**Table 3.** **The change from baseline to 30-days in PAR-ADP by BMI category**.

Point-in-time	Baseline		3 days		30 days	
BMI (kg/$m^{2}$)	≥24 (n = 67)	<24 (n = 38)	≥24 (n = 67)	<24 (n = 38)	≥24 (n = 67)	<24 (n = 38)
PAR-ADP (%)	65.58 ± 8.33	58.15 ± 9.50	7.51 ± 6.56	8.91 ± 8.98	14.17 ± 7.17	10.67 ± 7.41
*p*-value	0.009		0.672		0.005	

Abbreviations: BMI, body mass index; PAR-ADP, platelet aggregation rate induced by adenosine diphosphate.

### 3.3 Relationship between Body Weight and BMI, the Concentrations of Ticagrelor and AR-C124910XX, PAR-ADP, and IPA-ADP 

Three days after DAPT treatment, a negative correlation was observed between body weight and ticagrelor plasma concentrations (*r* = –0.276, *p* < 0.001) as well as its metabolite AR-C124910XX (*r* = –0.337, *p* < 0.001). Similar negative correlations were found when assessing BMI with ticagrelor (*r* = –0.173, *p* = 0.009) and AR-C124910XX (*r* = –0.207, *p* = 0.002). Interestingly, these negative correlations persisted 30 days after treatment. Body weight and BMI negatively correlated with the plasma concentrations of ticagrelor (*r* = –0.256, *p* < 0.001; *r* = –0.162, *p* = 0.015) as did its metabolite AR-C124910XX (*r* = –0.352, *p* < 0.001; *r* = –0.202, *p* = 0.002) (Table 4).

**Table 4.** **The relationship between body weight and BMI, the blood concentrations of ticagrelor and AR-C124910XX, PAR-ADP, and IPA-ADP**.

		Ticagrelor		AR-C124910XX		PAR-ADP			IPA-ADP	
		3 days	30 days	3 days	30 days	Baseline	3 days	30 days	3 days	30 days
Body weight	*r*	−0.276	−0.256	−0.337	−0.352	0.122	−0.032	0.171	0.029	−0.163
	*p*	<0.001	<0.001	<0.001	<0.001	0.022	0.637	0.010	0.060	0.015
BMI	*r*	−0.173	−0.162	−0.207	−0.202	0.205	0.010	0.217	−0.014	−0.211
	*p*	0.009	0.015	0.002	0.002	0.007	0.879	0.001	0.835	0.001

Abbreviations: BMI, body mass index; PAR-ADP, platelet aggregation rate induced by adenosine diphosphate; IPA-ADP, inhibition of platelet aggregation induced by adenosine diphosphate.

The correlation of body weight or BMI and PAR-ADP was positive at baseline (*r* = 0.122, *p* = 0.022; *r* = 0.205, *p* = 0.007), indicating that body weight and BMI affect platelet aggregation ability before DAPT treatment. There was no significant correlation between body weight or BMI and PAR-ADP or IPA-ADP 3 days after DAPT treatment (*p* > 0.05, refer to Table 4 for specific data).

However, there was a positive correlation between body weight and PAR-ADP (*r* = 0.171, *p* = 0.010), and a negative correlation between body weight and IPA-ADP (*r* = –0.163, *p* = 0.015) at 30 days after DAPT treatment. Furthermore, there was a positive correlation between BMI and PAR-ADP (*r* = 0.217, *p* = 0.001), and a negative correlation between BMI and IPA-ADP (*r* = –0.211, *p* = 0.001) at 30 days after DAPT treatment.

### 3.4 Correlation of the Concentrations of Ticagrelor and AR-C124910XX with PAR-ADP and IPA-ADP

There were no significant correlations between ticagrelor concentration and PAR-ADP (*r* = 0.016, *p* = 0.085) or IPA-ADP (*r* = –0.071, *p* = 0.284) after 3 days of DAPT treatment. Additionally, there were not correlations between the concentration of AR-C124910XX, PAR-ADP (*r* = 0.073, *p* = 0.276), or IPA-ADP (*r* = –0.073, *p* = 0.268), after 3 days of DAPT treatment (Table 5).

**Table 5.** **Relationship between the concentrations of ticagrelor and AR-C124910XX, PAR-ADP, and IPA-ADP at different time points after DAPT**.

		PAR-ADP		IPA-ADP	
		3 days	30 days	3 days	30 days
Ticagrelor	*r*	0.016	−0.355	−0.071	0.320
	*p*	0.805	<0.001	0.284	<0.001
AR-C124910XX	*r*	0.073	−0.226	−0.073	0.208
	*p*	0.276	0.001	0.268	0.002

Abbreviations: PAR-ADP, platelet aggregation rate induced by adenosine diphosphate; IPA-ADP, inhibition of platelet aggregation induced by adenosine diphosphate; DAPT, dual antiplatelet therapy.

Following 30 days of DAPT treatment, the plasma concentration of ticagrelor was negatively correlated with PAR-ADP (*r* = –0.335, *p* < 0.001) and positively correlated with IPA-ADP (*r* = 0.320, *p* < 0.001). Furthermore, there was also a negative correlation between AR-C124910XX concentration and PAR-ADP (*r* = –0.226, *p* = 0.001) and a positive correlation between the concentration of AR-C124910XX and IPA-ADP (*r* = 0.208, *p* = 0.002) at 30 days after treatment.

### 3.5 Incidence of MACE and Cox Multivariate Analysis

During a mean follow-up of 12 months, there were five MACE cases, including two stent stenoses, one ischemic stroke, and two clinically significant bleeding events. The two cases of clinically significant bleeding events, including one severe subcutaneous hemorrhage, and one gastrointestinal hemorrhage. To analyze both ischemic and bleeding events, factors including sex, age, hypertension, diabetes, smoking history, cerebrovascular history, and other comorbid conditions, as well as PAR-ADP, IPA-ADP, and the blood concentrations of ticagrelor and AR-C124910XX, were incorporated into Cox multivariate regression models. The ischemic events model showed a likelihood ratio (LR) of 22.886 (*p* = 0.153) and the Cox multivariate regression model produced a bleeding events LR of 8.881 (*p* = 0.944). However, these variables were not significantly associated with the occurrence of bleeding events or MACE within 12 months post-DAPT treatment, potentially due to the overall low incidence of MACEs.

## 4. Discussion

PAR measures the extent of platelet aggregation, while IPA assesses the effectiveness of treatments that prevent this aggregation. PAR-ADP, a specific index, gauges how well P2Y12 receptor inhibitors—a type of antiplatelet medication—are working. Previous studies have found that even after treating with clopidogrel, a P2Y12 inhibitor, high levels of PAR-ADP can persist [13, 14]. This is concerning because it is an independent predictor of ischemic events such as stent thrombosis and myocardial infarction one year after the initial PCI, despite the low risk of bleeding [13, 14]. In patients with coronary heart disease who underwent PCI, the combination of high baseline and post-treatment PARs was associated with a higher risk of recurrent ischemic events [15, 16]. Björklund *et al*. [17] found that severe bleeding events following ticagrelor treatment were associated with low PAR after that treatment. The SCORE study reported a positive correlation between BMI and platelet reactivity before treatment with P2Y12 receptor inhibitors [7]. Furthermore, patients with chronic coronary syndrome and higher BMI exhibited greater platelet reactivity even after receiving clopidogrel, a specific P2Y12 inhibitor [6]. This may be related to the relatively high basic platelet aggregation function and low response to antiplatelet aggregation therapy in patients with a higher BMI. Body weight and BMI may affect the metabolic concentration of antiplatelet aggregation drugs *in vivo * [18]. Our findings in this study further support this view. We found that both body weight and BMI were positively correlated with baseline PAR-ADP before DAPT treatment, and patients in the high BMI group (BMI ≥24 kg/$m^{2}$) had a higher PAR-ADP at baseline and 30 days after treatment with ticagrelor. In this study, we observed the PAR-ADP before and after DAPT treatment in patients with UA, and found that BMI and body weight were significant factors for the pharmacokinetics and pharmacodynamics of ticagrelor treatment.

In the pharmacokinetics study, we found significant negative correlations between body weight or BMI, and the concentrations of ticagrelor or its metabolite after 3 days and 30 days of treatment with DAPT, suggesting that body weight and BMI are important factors affecting ticagrelor metabolism. However, body weight and BMI had no significant correlation with PAR-ADP and IPA-ADP after 3 days of treatment with DAPT. Additionally, there were no correlations between the concentration of ticagrelor or its metabolite AR-C124910XX, and PAR-ADP or IPA-ADP at 3 days after DAPT treatment. However, after 30 days of DAPT treatment, when the only antithrombotic drugs were aspirin and ticagrelor, the correlation between plasma concentration of ticagrelor or AR-C12410XX and PAR-ADP was significant. These results may be explained by the fact that all the patients in this study underwent PCI. Patients with UA not only may be treated with antiplatelet aggregation drugs during the hospitalization, but also may be treated with heparin, low-molecular-weight heparin, and intravenous antiplatelet aggregation drugs (such as ${\mathrm{II}}_{\text{b}}$${\mathrm{III}}_{\text{a}}$ receptor antagonist tirofiban) before, during, and after their PCI. The body weight and BMI of the patients with relatively stable medications at 30 days were significantly correlated with PAR-ADP and IPA-ADP. These results indicate that body weight and BMI are still the significant factors affecting the metabolism and therapeutic effect of ticagrelor. In addition, we also found that sex was an influential factor for the pharmacodynamics and pharmacokinetics of ticagrelor treatment. Compared with males, females had higher blood concentrations of ticagrelor and its metabolites after treatment with ticagrelor. The PAR-ADP at 30 days after ticagrelor treatment was lower in female patients than that in males, which may be explained by the lower weight and BMI in the female patients.

The possible reasons for the reduced effectiveness of ticagrelor-based DAPT in inhibiting platelet aggregation in patients with higher body weight and BMI may be explained by two main factors. First, these patients typically have a more robust platelet aggregation function than that of patients with low body weight and BMI, resulting in a higher platelet aggregation rate after receiving DAPT. Second, body weight and BMI indirectly affected PAR and IPA after treatment by altering the serum concentrations of ticagrelor and its metabolite AR-C124910XX *in vivo*. However, our Cox multifactor survival analysis did not identify any significant influence of body weight, BMI, ticagrelor and AR-C124910XX concentrations, PAR-ADP, or IPA-ADP on the occurrence of bleeding or ischemic events in UA patients. These results may be related to the short follow-up time and low overall incidence of MACE in this study. In addition, prophylaxis with proton pump inhibitors in 80% of the study patients may have also reduced the risk of gastrointestinal bleeding with DAPT [19].

In summary, this study found that in patients with UA, BMI and body weight can affect the blood concentrations of ticagrelor and its metabolite after DAPT treatment, subsequently affecting PAR and IPA levels after treatment. An increase in body weight and BMI is associated with lower blood concentrations of ticagrelor after treatment and reduced responsiveness to DAPT. This suggests that the current dose of ticagrelor, 90 mg twice daily, may be too high in patients with low body weight and BMI and that a lower dose (e.g., 60 mg twice daily) may reduce the incidence of bleeding events without increasing the risk of ischemia [20]. The PEGASUS-TIMI study found that ticagrelor, 60 mg twice daily, reduced the incidence of MACEs and was not inferior to the standard 90 mg twice daily regimen [21]. However, a low-dose ticagrelor regimen can significantly reduce the risk of severe bleeding events following treatment with ticagrelor [12].

This study subject to certain limitations. First, the number of subjects included in our study is relatively small, which may impact the generalizability of the findings. Second, the overall study follow-up time was short, potentially limiting the observation of long-term effects. Despite these constraints, pilot study has confirmed that body weight and BMI are important factors affecting the metabolism and therapeutic effect of ticagrelor in patients with unstable angina pectoris after receiving ticagrelor treatment. These initial findings lay a solid foundation for future, larger-scale multicenter clinical studies aimed at comprehensively evaluating the impact of BMI and body weight on the long-term efficacy of DAPT.

## 5. Conclusions

In this study, we identified BMI and body weight as key factors influencing the pharmacokinetics and pharmacodynamics of ticagrelor in Chinese Han patients with UA undergoing DAPT treatment. We observed a positive correlation between BMI and body weight, which were positively correlated with PAR-ADP levels. This was evident at baseline and persisted for 30 days after DAPT treatment.

## Data Availability

The datasets generated or analyzed during this study are available from the corresponding author on reasonable request.

## References

[1] Wallentin L, Becker RC, Budaj A, Cannon CP, Emanuelsson H, Held C (2009). Ticagrelor versus clopidogrel in patients with acute coronary syndromes. *The New England Journal of Medicine*.

[2] Arora S, Shemisa K, Vaduganathan M, Qamar A, Gupta A, Garg SK (2019). Premature Ticagrelor Discontinuation in Secondary Prevention of Atherosclerotic CVD. *Journal of the American College of Cardiology*.

[3] Verdoia M, Savonitto S, Dudek D, Kedhi E, De Luca G (2021). Ticagrelor as compared to conventional antiplatelet agents in coronary artery disease: a comprehensive meta-analysis of 15 randomized trials. *Vascular Pharmacology*.

[4] Saito Y, Nishi T, Wakabayashi S, Ohno Y, Kitahara H, Ariyoshi N (2022). Differential Impact of Clinical and Genetic Factors on High Platelet Reactivity in Patients with Coronary Artery Disease Treated with Clopidogrel and Prasugrel. *Journal of Atherosclerosis and Thrombosis*.

[5] Parker WAE, Angiolillo DJ, Rollini F, Franchi F, Bonaca MP, Bhatt DL (2023). Influence of body weight and body mass index on the chronic pharmacokinetic and pharmacodynamic responses to clinically available doses of ticagrelor in patients with chronic coronary syndromes. *Vascular Pharmacology*.

[6] Puccini M, Rauch C, Jakobs K, Friebel J, Hassanein A, Landmesser U (2023). Being Overweight or Obese is Associated with an Increased Platelet Reactivity despite Dual Antiplatelet Therapy with Aspirin and Clopidogrel. *Cardiovascular Drugs and Therapy*.

[7] Ranucci M, Aloisio T, Di Dedda U, La Rovere MT, De Arroyabe BML, Baryshnikova E (2019). Platelet reactivity in overweight and obese patients undergoing cardiac surgery. *Platelets*.

[8] Liu S, Xue L, Shi X, Sun Z, Zhu Z, Zhang X (2018). Population pharmacokinetics and pharmacodynamics of ticagrelor and AR-C124910XX in Chinese healthy male subjects. *European Journal of Clinical Pharmacology*.

[9] Ntuk UE, Gill JMR, Mackay DF, Sattar N, Pell JP (2014). Ethnic-Specific Obesity Cutoffs for Diabetes Risk: Cross-sectional Study of 490,288 UK Biobank Participants. *Diabetes Care*.

[10] Collet JP, Thiele H, Barbato E, Barthélémy O, Bauersachs J, Bhatt DL (2021). 2020 ESC Guidelines for the management of acute coronary syndromes in patients presenting without persistent ST-segment elevation. *European Heart Journal*.

[11] Lawton JS, Tamis-Holland JE, Bangalore S, Bates ER, Beckie TM, Writing Committee Members (2022). 2021 ACC/AHA/SCAI Guideline for Coronary Artery Revascularization: Executive Summary: A Report of the American College of Cardiology/American Heart Association Joint Committee on Clinical Practice Guidelines. *Journal of the American College of Cardiology*.

[12] Kim L, Choe JC, Ahn JH, Lee HW, Oh JH, Choi JH (2021). Temporal Trends of Bleeding Episodes during Half- vs. Standard-Dose Ticagrelor in Acute Coronary Syndrome Patients with Low Platelet Reactivity: A Randomized BLEEDING-ACS Trial. *Journal of Clinical Medicine*.

[13] Li S, Feng X, Xu H, Chen K (2018). Comparison on Anticoagulation and Antiplatelet Aggregation Effects of Puerarin with Heparin Sodium and Tirofiban Hydrochloride: an in Vitro Study. *Chinese Journal of Integrative Medicine*.

[14] Zhou K, Yu S, Li J, Tan Y, Xing S, Chen Y (2022). High on‐treatment platelet reactivity is associated with poor outcomes after ischemic stroke: a meta‐analysis. *Acta Neurologica Scandinavica*.

[15] Choi SY, Kim MH, Serebruany V (2016). The challenge for predicting bleeding events by assessing platelet reactivity following coronary stenting. *International Journal of Cardiology*.

[16] Eyileten C, Gasecka A, Nowak A, Jarosz-Popek J, Wolska M, Dizdarevic A (2022). High concentration of symmetric dimethylarginine is associated with low platelet reactivity and increased bleeding risk in patients with acute coronary syndrome. *Thrombosis Research*.

[17] Björklund E, Hansson EC, Romlin BS, Jeppsson A, Malm CJ (2019). Postoperative platelet function is associated with severe bleeding in ticagrelor-treated patients. *Interactive CardioVascular and Thoracic Surgery*.

[18] Cho JY, Lee S, Yun KH, Kim B, Hong S, Ko JS (2021). Factors Related to Major Bleeding after Ticagrelor Therapy: Results from the TICO Trial. *Journal of the American Heart Association*.

[19] Khan MY, Siddiqui WJ, Alvarez C, Aggarwal S, Hasni SF, Ahmad A (2018). Reduction in postpercutaneous coronary intervention angina in addition to gastrointestinal events in patients on combined proton pump inhibitors and dual antiplatelet therapy: a systematic review and meta-analysis. *European Journal of Gastroenterology & Hepatology*.

[20] Sanderson NC, Parker WAE, Storey RF (2021). Ticagrelor: clinical development and future potential. *Reviews in Cardiovascular Medicine*.

[21] Bonaca MP, Bhatt DL, Cohen M, Steg PG, Storey RF, Jensen EC (2015). Long-Term Use of Ticagrelor in Patients with Prior Myocardial Infarction. *New England Journal of Medicine*.

